# Uso das Sementes de *Moringa Oleifera* no Tratamento da Água

**DOI:** 10.36660/abc.20200390

**Published:** 2020-06-29

**Authors:** Marcia Kiyomi Koike, Akimi Kokanj Kochi, Denise Yamada Gomes Pinto

**Affiliations:** 1 Departamento de Clínica Médica Faculdade de Medicina Universidade de São Paulo São Paulo Brasil Laboratório de Emergências Clínicas - Departamento de Clínica Médica - Faculdade de Medicina da Universidade de São Paulo, São Paulo, Brasil; 2 Programa de Pós-graduação em Ciências da Saúde Instituto de Assistência ao Servidor Público Estadual de São Paulo São PauloSP Brasil Programa de Pós-graduação em Ciências da Saúde - Instituto de Assistência ao Servidor Público Estadual de São Paulo (IAMSPE), São Paulo, SP – Brasil

**Keywords:** Moringa Oleífera, Glicosídeos, Anti-Inflamatórios, Lectinas de Plantas, Segurança Hídrica

A *Moringa oleifera* Lamarck (MO) é uma planta da família das *Moringaceae* , nativa dos Himalaias e que se adaptou nos diversos continentes, sendo amplamente cultivada na Ásia, África e Américas.^1^ De crescimento rápido, a planta toda tem uma grande variedade de aplicações na dieta e na medicina popular.

No entanto, evidências científicas de suas propriedades começaram a surgir apenas no início de 2000. Em estudos experimentais *in vitro* ou *ex-vivo* , as folhas e sementes apresentaram diversos efeitos biológicos, como anti-inflamatórios e cicatrizantes,^2^ antitumorais,^3^ antidiabético,^4^ antioxidante^4 , 5^ e na função sexual.^6 , 7^

Devido suas propriedades de flotação e ação antimicrobiana, as sementes têm sido utilizadas na purificação da água.^8^ Trata-se de um método de baixo custo, que faz uso de recursos naturais e de fácil manuseio, que pode oferecer qualidade na água das comunidades pobres. As sementes podem adsorver poluentes como herbicidas,^9^ metais pesados,^10^ medicamentos^11 , 12^ e atuar como larvicidas e antimicrobianos natural.^13^

Entre os componentes presentes na semente de *Moringa oleifera* , a lectina solúvel em água (WSMoL) apresenta a propriedade de larvicida e ovicida do *Aedes aegypti* ^14^ e antinematódeos.^15^

Como todo conhecimento científico sobre a *Moringa oleifera* ainda está baseado em estudos experimentais, há necessidade de respeitar as etapas da pesquisa clínica para uso em humanos. Assim, em 2019, a Agência Nacional de Vigilância Sanitária (ANVISA) proibiu a fabricação, importação, comercialização, propaganda e distribuição de todos os alimentos que contenham *Moringa oleifera (RESOLUÇÃO-RE Nº 1.478, DE 3 DE JUNHO DE 2019).*

Da mesma forma, em relação ao uso das sementes para purificação de água, um estudo para se certificar da segurança também faz necessário. Neste contexto, nesta edição dos Arquivos Brasileiros de Cardiologia, Yurre et al.^16^ conduziram uma cuidadosa investigação sobre os efeitos cardiotóxicos da WSMoL de sementes de *Moringa oleifera* . Os autores avaliaram os possíveis efeitos celulares, estruturais, elétricos e funcionais no coração; bem como efeitos no metabolismo de carboidratos e no peso corporal. O estudo buscou testar sua hipótese nula e teve êxito em demonstrar, experimentalmente, a segurança de utilização da WSMoL por 21 dias, e estimula novos projetos de avaliação da segurança do uso de sementes de *Moringa oleifera* para a purificação da água para o uso humano.
