# Supplementary material for: Uso das Sementes de Moringa Oleifera no Tratamento da Água
Source: Arq Bras Cardiol. 2020 Jun 29;114(6):1038–9. [Article in Portuguese] doi: 10.36660/abc.20200390 (PMC8416126; doi:10.36660/abc.20200390)
Supplement: Translation

# Translation: Use of Moringa Oleifera Seeds in Water Treatment

**Authors:** Marcia Kiyomi Koike, Akimi Kokanj Kochi, Denise Yamada Gomes Pinto

**Affiliations:** 1 Brazil Laboratório de Emergências Clínicas - Departamento de Clínica Médica - Faculdade de Medicina da Universidade de São Paulo, São Paulo, Brazil; 2 Brazil Programa de Pós-graduação em Ciências da Saúde - Instituto de Assistência ao Servidor Público Estadual de São Paulo (IAMSPE), São Paulo, SP – Brazil

**Keywords:** Moringa Oleifera, Glycosides, Anti-Inflammatory Agents, Plant Lectins, Water Security

*Moringa oleifera* Lamarck (MO) is a plant of the *Moringaceae* family, native to the Himalayas and adapted on several continents, is widely cultivated in Asia, Africa and the Americas.^1^ Rapidly growing, the whole plant has a wide variety of applications in diet and folk medicine.

However, scientific evidence of its properties began to emerge only in the beginning of 2000. In experimental studies *in vitro* or *ex-vivo* , leaves and seeds showed several biological effects, such as anti-inflammatory and wound healing,^2^ antitumor,^3^ antidiabetic,^4^ antioxidant^4 , 5^ and sexual function.^6 , 7^

Due to their flotation properties and antimicrobial action, seeds have been used to purify water.^8^ It is a low-cost method, which uses natural resources and easy handling that can offer quality in the water of poor communities. The seeds can absorb pollutants such as herbicides,^9^ heavy metals,^10^ medications^11 , 12^ and act as larvicides and natural antimicrobials.^13^

Among the components present in *Moringa oleifera* seed, water-soluble lectin (WSMoL) has the property of *Aedes aegypti* larvicide and ovicide^14^ and anti nematodes.^15^

As all scientific knowledge about *Moringa oleifera* is still based on experimental studies, there is a need to respect the stages of clinical research for use in humans. Thus, in 2019 in Brazil, the National Health Surveillance Agency (ANVISA) prohibited the manufacture, import, marketing, advertising and distribution of all foods containing *Moringa oleifera* (RESOLUTION-RE No. 1,478, OF 3 JUNE 2019)

Likewise, in relation to the use of seeds for water purification, a study to ensure safety is also necessary. In this context, in this edition of the *Arquivos Brasileiros de Cardiologia* , Yurre et al.^16^ conducted a careful investigation of the cardiotoxic effects of WSMoL from *Moringa oleifera* seeds. The authors evaluated the possible cellular, structural, electrical and functional effects on the heart, effects on carbohydrate metabolism and body weight. It was a study that sought to test its null hypothesis and was successful in demonstrating, experimentally, the safety of using WSMoL for 21 days and it encourages new projects to evaluate the safety of using *Moringa oleifer* a seeds for the purification of water for human use.
